# Diaqua­bis(4-methyl­amino­benzoato-κ*O*)bis­(nicotinamide-κ*N* ^1^)cobalt(II)

**DOI:** 10.1107/S1600536810010706

**Published:** 2010-03-27

**Authors:** Hacali Necefoğlu, Özgür Aybirdi, Barış Tercan, Emel Ermiş, Tuncer Hökelek

**Affiliations:** a Department of Chemistry, Kafkas University, 36100 Kars, Turkey; b Department of Physics, Karabük University, 78050 Karabük, Turkey; c Department of Chemistry, Faculty of Science, Anadolu University, 26470 Yenibağlar, Eskişehir, Turkey; d Department of Physics, Hacettepe University, 06800 Beytepe, Ankara, Turkey

## Abstract

The asymmetric unit of the title Co^II^ complex, [Co(C_8_H_8_NO_2_)_2_(C_6_H_6_N_2_O)_2_(H_2_O)_2_], contains two half complex mol­ecules with similar structures. The Co^II^ atoms are each located on an inversion center and each is coordinated by two 4-methyl­amino­benzoate (PMAB), two nicotinamide (NA) ligands and two water mol­ecules in a distorted octa­hedral coordination. The dihedral angles between the carboxyl­ate groups and the adjacent benzene rings are 3.0 (3) and 2.54 (19)°, while the pyridine and benzene rings are oriented at dihedral angles of 67.40 (8) and 66.25 (8)°. In the crystal structure, inter­molecular O—H⋯O and N—H⋯O hydrogen bonds link the mol­ecules into a supra­molecular structure.

## Related literature

For niacin, see: Krishnamachari (1974 ▶) and for the nicotinic acid derivative *N*,*N*-diethyl­nicotinamide, see: Bigoli *et al.* (1972 ▶). For related structures, see: Hökelek *et al.* (1996 ▶); Hökelek & Necefoğlu (1998 ▶); Hökelek *et al.* (2009*a* ▶,*b* ▶,*c* ▶).
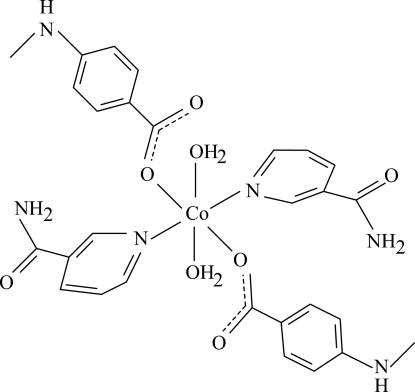


## Experimental

### 

#### Crystal data

[Co(C_8_H_8_NO_2_)_2_(C_6_H_6_N_2_O)_2_(H_2_O)_2_]
*M* *_r_* = 639.53
Triclinic, 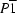

*a* = 9.9014 (7) Å
*b* = 11.2891 (8) Å
*c* = 14.1824 (9) Å
α = 107.554 (5)°
β = 92.975 (4)°
γ = 92.836 (4)°
*V* = 1505.89 (18) Å^3^
*Z* = 2
Mo *K*α radiation
μ = 0.63 mm^−1^
*T* = 294 K
0.31 × 0.14 × 0.11 mm

#### Data collection

Bruker Kappa APEXII CCD area-detector diffractometer
Absorption correction: multi-scan (*SADABS*; Bruker, 2005 ▶) *T*_min_ = 0.889, *T*_max_ = 0.934
25128 measured reflections
7337 independent reflections
3927 reflections with *I* > 2σ(*I*)
*R*_int_ = 0.050

#### Refinement

*R*[*F* ^2^ > 2σ(*F* ^2^)] = 0.047
*wR*(*F* ^2^) = 0.120
*S* = 0.99
7337 reflections
433 parameters
11 restraints
H atoms treated by a mixture of independent and constrained refinement
Δρ_max_ = 0.44 e Å^−3^
Δρ_min_ = −0.64 e Å^−3^

### 

Data collection: *APEX2* (Bruker, 2007 ▶); cell refinement: *SAINT* (Bruker, 2007 ▶); data reduction: *SAINT*; program(s) used to solve structure: *SHELXS97* (Sheldrick, 2008 ▶); program(s) used to refine structure: *SHELXL97* (Sheldrick, 2008 ▶); molecular graphics: *Mercury* (Macrae *et al.*, 2006 ▶); software used to prepare material for publication: *WinGX* (Farrugia, 1999 ▶).

## Supplementary Material

Crystal structure: contains datablocks I, global. DOI: 10.1107/S1600536810010706/xu2735sup1.cif

Structure factors: contains datablocks I. DOI: 10.1107/S1600536810010706/xu2735Isup2.hkl

Additional supplementary materials:  crystallographic information; 3D view; checkCIF report

## Figures and Tables

**Table 1:** Selected bond lengths (Å)

Co1—O1	2.0553 (17)
Co1—O4	2.088 (2)
Co1—N2	2.153 (2)
Co2—O5	2.0696 (18)
Co2—O8	2.129 (2)
Co2—N5	2.159 (2)

**Table 2:** Hydrogen-bond geometry (Å, °)

*D*—H⋯*A*	*D*—H	H⋯*A*	*D*⋯*A*	*D*—H⋯*A*
O4—H41⋯O7^i^	0.88 (2)	1.75 (2)	2.618 (4)	173 (4)
O4—H42⋯O2^ii^	0.89 (4)	1.86 (4)	2.644 (3)	146 (4)
O8—H81⋯O3^iii^	0.91 (2)	1.84 (2)	2.742 (3)	173 (3)
O8—H82⋯O6^iv^	0.94 (3)	1.76 (3)	2.648 (3)	156 (3)
N3—H32⋯O2^v^	0.91 (3)	2.04 (3)	2.939 (4)	168 (3)
N4—H4*A*⋯O4^v^	0.88 (4)	2.53 (4)	3.288 (4)	145 (3)
N6—H61⋯O6^vi^	0.90 (3)	2.08 (3)	2.973 (4)	169 (3)
N6—H62⋯O3^i^	0.88 (3)	2.36 (3)	3.124 (4)	146 (3)

Symmetry codes: (i) 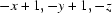; (ii) 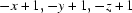; (iii) 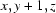; (iv) 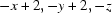; (v) 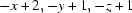; (vi) 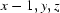.

**Fig. 1.**
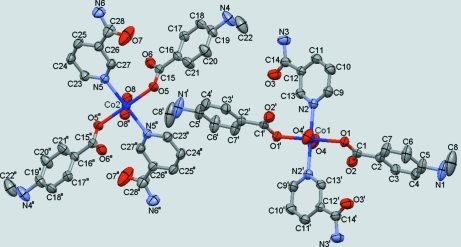
The molecular structure of the title molecule with the atom-numbering scheme. Displacement ellipsoids are drawn at the 50% probability level. Primed atoms are generated by the symmetry operators:(') 1 - x, 1 - y, 1 - z; ('') 2 - x, 2 - y, -z.

## supplementary crystallographic information

### Comment

As a part of our ongoing investigation on transition metal complexes of nicotinamide (NA), one form of niacin (Krishnamachari, 1974), and/or the nicotinic acid derivative *N*,*N*-diethylnicotinamide (DENA), an important respiratory stimulant (Bigoli *et al.*, 1972), the title compound was synthesized and its crystal structure is reported herein.

The asymmetric unit of the title complex, (I), contains two half molecules with Co atoms on inversion centers. Each molecule contains two 4-methylaminobenzoate (PMAB) and two nicotinamide (NA) ligands and two coordinated water molecules, all ligands being monodentate. The crystal structures of similar complexes of Cu^II^, Co^II^, Ni^II^, Mn^II^ and Zn^II^ ions, [Cu(C_7_H_5_O_2_)_2_(C_10_H_14_N_2_O)_2_], (II) (Hökelek *et al.*, 1996), [Co(C_6_H_6_N_2_O)_2_(C_7_H_4_NO_4_)_2_(H_2_O)_2_], (III) (Hökelek & Necefoğlu, 1998), [Ni(C_7_H_4_ClO_2_)_2_(C_6_H_6_N_2_O)_2_(H_2_O)_2_], (IV) (Hökelek *et al.*, 2009a), [Mn(C_7_H_4_ClO_2_)_2_(C_10_H_14_N_2_O)_2_(H_2_O)_2_], (V) (Hökelek *et al.*, 2009b) and [Zn(C_7_H_4_BrO_2_)_2_(C_6_H_6_N_2_O)_2_(H_2_O)_2_], (VI) (Hökelek *et al.*, 2009c) have also been reported. In (II), the two benzoate ions are coordinated to the Cu atom as bidentate ligands, while in the other structures all ligands being monodentate.

Each of the two molecules in the asymmetric unit of the title complex, [Co(PMAB)_2_(NA)_2_(H_2_O)_2_], has a centre of symmetry and Co^II^ ion is surrounded by two PMAB and two NA ligands and two water molecules (Fig. 1). All ligands are monodentate. In each molecule, the four O atoms (O1, O4, O1', O4' and O5, O8, O5'', o8'') in the equatorial plane around the Co atom form a slightly distorted square-planar arrangement, while the slightly distorted octahedral coordination is completed by the two N atoms of the NA ligands (N2, N2' and N5, N5'') in the axial positions (Fig. 1).

The near equality of the C1—O1 [1.274 (3) Å], C1—O2 [1.255 (3) Å] and C15—O5 [1.276 (3) Å], C15—O6 [1.262 (3) Å] bonds in the carboxylate groups indicate delocalized bonding arrangements, rather than localized single and double bonds. The average Co—O bond lengths are 2.0717 (19) and 2.0993 (19) Å (Table 1) and the Co atoms are displaced out of the least-squares planes of the carboxylate groups [(O1/C1/O2) and (O5/C15/O6)] by -0.4948 (1) and -0.3495 (1) Å, respectively. The dihedral angles between the planar carboxylate groups and the benzene rings A (C2—C7) and C (C16—C21) are 2.96 (27)° and 2.54 (19)°, respectively, while that between rings A and B (N2/C9—C13) and C and D (N5/C23—C27) are 67.40 (8) and 66.25 (8)°, respectively.

In the crystal structure, intermolecular O—H···O and N—H···O hydrogen bonds (Table 2) link the molecules into a supramolecular structure, in which they may be effective in the stabilization of the structure.

### Experimental

The title compound was prepared by the reaction of CoSO_4_.7(H_2_O) (1.41 g, 5 mmol) in H_2_O (30 ml) and NA (1.22 g, 10 mmol) in H_2_O (20 ml) with sodium 4-methylaminobenzoate (1.74 g, 10 mmol) in H_2_O (50 ml). The mixture was filtered and set aside to crystallize at ambient temperature for one week, giving orange single crystals.

### Refinement

Atoms H1, H4A (for NH), H31, H32, H61, H62 (for NH_2_) and H41, H42, H81, H82 (for H_2_O) were located in a difference Fourier map and refined isotropically, with restrains of N3—H31 = 0.870 (19), N3—H32 = 0.909 (19), N4—H4A = 0.87 (2), N6—H61 = 0.904 (19), N6—H62 = 0.878 (19), O4—H41 = 0.876 (17), O4—H42 = 0.89 (4), O8—H81 = 0.907 (14), O8—H82 = 0.932 (18) Å and H41—O4—H42 = 106 (3) and H81—O8—H82 = 106 (2)°. The remaining H atoms were positioned geometrically with C—H = 0.93 and 0.96 Å, for aromatic and methyl H atoms, respectively, and constrained to ride on their parent atoms, with U_iso_(H) = xU_eq_(C), where x = 1.5 for methyl H and x = 1.2 for aromatic H atoms.

### Crystal data

**Table:**

[Co(C_8_H_8_NO_2_)_2_(C_6_H_6_N_2_O)_2_(H_2_O)_2_]	*Z* = 2
*M**_r_* = 639.53	*F*(000) = 666
Triclinic, *P*1	*D*_x_ = 1.410 Mg m^−^^3^
Hall symbol: -P 1	Mo *K*α radiation, λ = 0.71073 Å
*a* = 9.9014 (7) Å	Cell parameters from 4808 reflections
*b* = 11.2891 (8) Å	θ = 2.5–22.9°
*c* = 14.1824 (9) Å	µ = 0.63 mm^−^^1^
α = 107.554 (5)°	*T* = 294 K
β = 92.975 (4)°	Block, orange
γ = 92.836 (4)°	0.31 × 0.14 × 0.11 mm
*V* = 1505.89 (18) Å^3^	

### Data collection

**Table:**

Bruker Kappa APEXII CCD area-detector diffractometer	7337 independent reflections
Radiation source: fine-focus sealed tube	3927 reflections with *I* > 2σ(*I*)
graphite	*R*_int_ = 0.050
φ and ω scans	θ_max_ = 28.3°, θ_min_ = 1.5°
Absorption correction: multi-scan (*SADABS*; Bruker, 2005)	*h* = −10→13
*T*_min_ = 0.889, *T*_max_ = 0.934	*k* = −14→15
25128 measured reflections	*l* = −18→18

### Refinement

**Table:**

Refinement on *F*^2^	Primary atom site location: structure-invariant direct methods
Least-squares matrix: full	Secondary atom site location: difference Fourier map
*R*[*F*^2^ > 2σ(*F*^2^)] = 0.047	Hydrogen site location: inferred from neighbouring sites
*wR*(*F*^2^) = 0.120	H atoms treated by a mixture of independent and constrained refinement
*S* = 0.99	*w* = 1/[σ^2^(*F*_o_^2^) + (0.0507*P*)^2^] where *P* = (*F*_o_^2^ + 2*F*_c_^2^)/3
7337 reflections	(Δ/σ)_max_ < 0.001
433 parameters	Δρ_max_ = 0.44 e Å^−^^3^
11 restraints	Δρ_min_ = −0.64 e Å^−^^3^

### Special details

**Table:**

Geometry. All esds (except the esd in the dihedral angle between two l.s. planes) are estimated using the full covariance matrix. The cell esds are taken into account individually in the estimation of esds in distances, angles and torsion angles; correlations between esds in cell parameters are only used when they are defined by crystal symmetry. An approximate (isotropic) treatment of cell esds is used for estimating esds involving l.s. planes.
Refinement. Refinement of *F*^2^ against ALL reflections. The weighted *R*-factor wR and goodness of fit *S* are based on *F*^2^, conventional *R*-factors *R* are based on *F*, with *F* set to zero for negative *F*^2^. The threshold expression of *F*^2^ > σ(*F*^2^) is used only for calculating *R*-factors(gt) etc. and is not relevant to the choice of reflections for refinement. *R*-factors based on *F*^2^ are statistically about twice as large as those based on *F*, and *R*- factors based on ALL data will be even larger.

### Fractional atomic coordinates and isotropic or equivalent isotropic displacement parameters (Å^2^)

**Table:**

	*x*	*y*	*z*	*U*_iso_*/*U*_eq_	
Co1	0.5000	0.5000	0.5000	0.03935 (17)	
Co2	1.0000	1.0000	0.0000	0.03728 (17)	
O1	0.56594 (18)	0.56020 (18)	0.38612 (14)	0.0449 (5)	
O2	0.71996 (19)	0.7140 (2)	0.46562 (16)	0.0532 (6)	
O3	0.8902 (2)	0.2578 (2)	0.27847 (16)	0.0663 (7)	
O4	0.4510 (3)	0.3195 (2)	0.40609 (19)	0.0673 (7)	
H41	0.447 (4)	0.285 (3)	0.3417 (13)	0.095 (14)*	
H42	0.383 (3)	0.283 (4)	0.428 (3)	0.135 (18)*	
O5	1.07631 (18)	0.95050 (19)	0.12042 (14)	0.0472 (5)	
O6	1.1978 (2)	0.7922 (2)	0.04475 (15)	0.0528 (6)	
O7	0.5387 (3)	0.7866 (4)	−0.21529 (18)	0.1282 (14)	
O8	0.98305 (19)	1.1857 (2)	0.09213 (16)	0.0472 (5)	
H81	0.958 (3)	1.207 (3)	0.1553 (12)	0.083 (12)*	
H82	0.918 (3)	1.215 (3)	0.056 (2)	0.104 (15)*	
N1	0.8652 (4)	0.6271 (3)	0.0185 (3)	0.0932 (13)	
H1	0.931 (4)	0.680 (4)	0.027 (3)	0.101 (16)*	
N2	0.7005 (2)	0.4443 (2)	0.52402 (18)	0.0444 (6)	
N3	1.0849 (3)	0.2554 (3)	0.3640 (2)	0.0585 (8)	
H31	1.122 (3)	0.222 (3)	0.3086 (17)	0.072 (12)*	
H32	1.135 (3)	0.264 (3)	0.4217 (18)	0.075 (12)*	
N4	1.3862 (4)	0.8673 (3)	0.4966 (2)	0.0738 (9)	
H4A	1.441 (4)	0.808 (3)	0.495 (3)	0.125 (19)*	
N5	0.7987 (2)	0.9412 (2)	0.02697 (17)	0.0395 (6)	
N6	0.3613 (3)	0.7723 (3)	−0.1287 (2)	0.0589 (8)	
H61	0.322 (3)	0.782 (4)	−0.0709 (19)	0.097 (15)*	
H62	0.313 (3)	0.739 (3)	−0.1852 (18)	0.094 (14)*	
C1	0.6647 (3)	0.6378 (3)	0.3879 (2)	0.0394 (7)	
C2	0.7153 (3)	0.6358 (3)	0.2905 (2)	0.0407 (7)	
C3	0.8201 (3)	0.7192 (3)	0.2835 (2)	0.0469 (8)	
H3	0.8571	0.7793	0.3407	0.056*	
C4	0.8694 (3)	0.7144 (3)	0.1946 (3)	0.0557 (9)	
H4	0.9404	0.7704	0.1925	0.067*	
C5	0.8157 (4)	0.6279 (3)	0.1075 (3)	0.0608 (9)	
C6	0.7106 (4)	0.5444 (3)	0.1136 (2)	0.0702 (11)	
H6	0.6731	0.4847	0.0564	0.084*	
C7	0.6619 (3)	0.5501 (3)	0.2042 (2)	0.0581 (9)	
H7	0.5910	0.4943	0.2068	0.070*	
C8	0.8172 (6)	0.5427 (5)	−0.0756 (3)	0.130 (2)	
H8A	0.8674	0.5600	−0.1266	0.194*	
H8B	0.7227	0.5524	−0.0880	0.194*	
H8C	0.8290	0.4588	−0.0756	0.194*	
C9	0.7586 (3)	0.4567 (3)	0.6138 (2)	0.0510 (8)	
H9	0.7126	0.4949	0.6694	0.061*	
C10	0.8847 (3)	0.4149 (3)	0.6270 (2)	0.0586 (9)	
H10	0.9228	0.4254	0.6905	0.070*	
C11	0.9534 (3)	0.3580 (3)	0.5463 (2)	0.0504 (8)	
H11	1.0387	0.3300	0.5544	0.060*	
C12	0.8947 (3)	0.3427 (3)	0.4525 (2)	0.0372 (7)	
C13	0.7679 (3)	0.3875 (3)	0.4461 (2)	0.0427 (7)	
H13	0.7273	0.3771	0.3833	0.051*	
C14	0.9565 (3)	0.2822 (3)	0.3585 (2)	0.0434 (7)	
C15	1.1621 (3)	0.8704 (3)	0.1220 (2)	0.0412 (7)	
C16	1.2203 (3)	0.8699 (3)	0.2201 (2)	0.0392 (7)	
C17	1.3176 (3)	0.7886 (3)	0.2286 (2)	0.0444 (7)	
H17	1.3464	0.7329	0.1716	0.053*	
C18	1.3718 (3)	0.7889 (3)	0.3193 (2)	0.0506 (8)	
H18	1.4372	0.7338	0.3227	0.061*	
C19	1.3313 (3)	0.8698 (3)	0.4065 (2)	0.0512 (8)	
C20	1.2335 (3)	0.9511 (3)	0.3987 (2)	0.0610 (9)	
H20	1.2043	1.0065	0.4556	0.073*	
C21	1.1798 (3)	0.9499 (3)	0.3069 (2)	0.0542 (8)	
H21	1.1141	1.0046	0.3033	0.065*	
C22	1.3480 (4)	0.9441 (4)	0.5901 (3)	0.0900 (13)	
H22A	1.3968	0.9234	0.6426	0.135*	
H22B	1.2524	0.9305	0.5946	0.135*	
H22C	1.3693	1.0300	0.5959	0.135*	
C23	0.7582 (3)	0.9468 (3)	0.1165 (2)	0.0473 (8)	
H23	0.8186	0.9812	0.1716	0.057*	
C24	0.6309 (3)	0.9038 (3)	0.1305 (2)	0.0555 (9)	
H24	0.6069	0.9080	0.1939	0.067*	
C25	0.5397 (3)	0.8547 (3)	0.0506 (2)	0.0500 (8)	
H25	0.4525	0.8269	0.0590	0.060*	
C26	0.5796 (3)	0.8473 (3)	−0.0430 (2)	0.0413 (7)	
C27	0.7095 (3)	0.8909 (3)	−0.0501 (2)	0.0423 (7)	
H27	0.7370	0.8850	−0.1130	0.051*	
C28	0.4910 (3)	0.7982 (3)	−0.1358 (2)	0.0597 (9)	

### Atomic displacement parameters (Å^2^)

**Table:**

	*U*^11^	*U*^22^	*U*^33^	*U*^12^	*U*^13^	*U*^23^
Co1	0.0366 (3)	0.0502 (4)	0.0358 (3)	0.0086 (3)	0.0118 (2)	0.0174 (3)
Co2	0.0287 (3)	0.0528 (4)	0.0361 (3)	0.0039 (2)	0.0022 (2)	0.0220 (3)
O1	0.0377 (11)	0.0619 (13)	0.0409 (12)	−0.0003 (10)	0.0061 (9)	0.0244 (11)
O2	0.0475 (13)	0.0688 (15)	0.0435 (13)	−0.0024 (11)	0.0024 (10)	0.0185 (12)
O3	0.0491 (13)	0.1049 (19)	0.0384 (13)	0.0182 (13)	−0.0002 (11)	0.0104 (13)
O4	0.0795 (18)	0.0634 (16)	0.0509 (17)	−0.0046 (13)	0.0278 (14)	0.0030 (14)
O5	0.0372 (11)	0.0688 (14)	0.0457 (13)	0.0121 (10)	0.0036 (9)	0.0312 (11)
O6	0.0536 (13)	0.0680 (15)	0.0419 (13)	0.0137 (11)	0.0028 (11)	0.0229 (12)
O7	0.091 (2)	0.226 (4)	0.0340 (15)	−0.069 (2)	0.0089 (14)	0.001 (2)
O8	0.0396 (12)	0.0611 (14)	0.0403 (13)	0.0042 (10)	0.0031 (10)	0.0140 (12)
N1	0.137 (3)	0.079 (3)	0.060 (2)	−0.028 (2)	0.047 (2)	0.015 (2)
N2	0.0420 (14)	0.0562 (16)	0.0387 (15)	0.0089 (12)	0.0067 (12)	0.0188 (13)
N3	0.0435 (17)	0.078 (2)	0.048 (2)	0.0220 (15)	0.0064 (16)	0.0084 (17)
N4	0.107 (3)	0.070 (2)	0.0462 (19)	0.017 (2)	−0.0184 (18)	0.0227 (17)
N5	0.0330 (13)	0.0517 (15)	0.0356 (14)	0.0027 (11)	0.0041 (11)	0.0158 (12)
N6	0.0455 (18)	0.073 (2)	0.049 (2)	−0.0099 (15)	−0.0066 (16)	0.0087 (17)
C1	0.0327 (16)	0.0487 (19)	0.0444 (19)	0.0144 (14)	0.0059 (14)	0.0233 (16)
C2	0.0422 (17)	0.0439 (18)	0.0419 (18)	0.0071 (14)	0.0093 (14)	0.0204 (15)
C3	0.0459 (18)	0.0496 (19)	0.0495 (19)	−0.0011 (14)	0.0016 (15)	0.0224 (16)
C4	0.054 (2)	0.061 (2)	0.060 (2)	−0.0080 (16)	0.0127 (17)	0.0312 (19)
C5	0.084 (3)	0.052 (2)	0.052 (2)	−0.0005 (18)	0.0278 (19)	0.0217 (18)
C6	0.105 (3)	0.055 (2)	0.045 (2)	−0.020 (2)	0.022 (2)	0.0087 (18)
C7	0.068 (2)	0.055 (2)	0.050 (2)	−0.0122 (17)	0.0185 (17)	0.0154 (18)
C8	0.215 (6)	0.108 (4)	0.057 (3)	−0.038 (4)	0.054 (3)	0.012 (3)
C9	0.052 (2)	0.066 (2)	0.0403 (19)	0.0103 (16)	0.0120 (16)	0.0213 (17)
C10	0.054 (2)	0.083 (3)	0.041 (2)	0.0130 (18)	−0.0018 (16)	0.0220 (19)
C11	0.0420 (18)	0.065 (2)	0.047 (2)	0.0130 (15)	−0.0002 (15)	0.0199 (17)
C12	0.0331 (15)	0.0448 (17)	0.0357 (17)	0.0051 (13)	0.0026 (13)	0.0149 (14)
C13	0.0405 (17)	0.057 (2)	0.0330 (17)	0.0088 (14)	0.0019 (14)	0.0163 (15)
C14	0.0361 (17)	0.0513 (19)	0.0422 (19)	0.0084 (14)	0.0035 (15)	0.0123 (16)
C15	0.0321 (16)	0.053 (2)	0.0464 (19)	−0.0002 (14)	0.0020 (14)	0.0266 (17)
C16	0.0391 (16)	0.0417 (17)	0.0414 (18)	0.0016 (13)	−0.0041 (14)	0.0208 (15)
C17	0.0459 (17)	0.0493 (19)	0.0418 (18)	0.0056 (14)	0.0030 (14)	0.0193 (15)
C18	0.0485 (19)	0.053 (2)	0.056 (2)	0.0137 (15)	−0.0057 (16)	0.0263 (18)
C19	0.062 (2)	0.050 (2)	0.044 (2)	0.0020 (16)	−0.0131 (17)	0.0217 (17)
C20	0.084 (3)	0.057 (2)	0.041 (2)	0.0205 (19)	−0.0042 (18)	0.0108 (17)
C21	0.064 (2)	0.051 (2)	0.049 (2)	0.0187 (16)	−0.0065 (17)	0.0167 (17)
C22	0.134 (4)	0.087 (3)	0.046 (2)	0.007 (3)	−0.020 (2)	0.021 (2)
C23	0.0425 (18)	0.065 (2)	0.0356 (17)	−0.0014 (15)	−0.0003 (14)	0.0192 (16)
C24	0.0474 (19)	0.088 (3)	0.0362 (18)	−0.0034 (18)	0.0104 (15)	0.0261 (18)
C25	0.0361 (17)	0.068 (2)	0.0447 (19)	−0.0073 (15)	0.0085 (15)	0.0170 (17)
C26	0.0357 (16)	0.0489 (18)	0.0381 (17)	−0.0016 (13)	0.0039 (13)	0.0120 (15)
C27	0.0402 (17)	0.0529 (19)	0.0339 (17)	0.0028 (14)	0.0089 (14)	0.0125 (15)
C28	0.055 (2)	0.071 (2)	0.043 (2)	−0.0177 (18)	0.0018 (17)	0.0048 (18)

### Geometric parameters (Å, °)

**Table:**

Co1—O1	2.0553 (17)	C3—H3	0.9300
Co1—O1^i^	2.0553 (17)	C4—C5	1.384 (4)
Co1—O4	2.088 (2)	C4—H4	0.9300
Co1—O4^i^	2.088 (2)	C5—C6	1.392 (4)
Co1—N2	2.153 (2)	C6—C7	1.381 (4)
Co1—N2^i^	2.153 (2)	C6—H6	0.9300
Co2—O5	2.0696 (18)	C7—H7	0.9300
Co2—O5^ii^	2.0696 (18)	C8—H8A	0.9600
Co2—O8	2.129 (2)	C8—H8B	0.9600
Co2—O8^ii^	2.129 (2)	C8—H8C	0.9600
Co2—N5	2.159 (2)	C9—H9	0.9300
Co2—N5^ii^	2.159 (2)	C10—C9	1.380 (4)
O1—C1	1.274 (3)	C10—H10	0.9300
O2—C1	1.255 (3)	C11—C10	1.366 (4)
O3—C14	1.229 (3)	C11—H11	0.9300
O4—H41	0.876 (17)	C12—C11	1.381 (4)
O4—H42	0.89 (4)	C12—C14	1.481 (4)
O5—C15	1.276 (3)	C13—C12	1.385 (4)
O6—C15	1.262 (3)	C13—H13	0.9300
O7—C28	1.218 (4)	C15—C16	1.479 (4)
O8—H81	0.907 (14)	C16—C17	1.389 (4)
O8—H82	0.932 (18)	C16—C21	1.382 (4)
N1—C5	1.376 (4)	C17—C18	1.365 (4)
N1—C8	1.428 (5)	C17—H17	0.9300
N1—H1	0.84 (4)	C18—C19	1.389 (4)
N2—C9	1.335 (3)	C18—H18	0.9300
N2—C13	1.330 (3)	C19—C20	1.389 (4)
N3—C14	1.325 (4)	C20—C21	1.375 (4)
N3—H31	0.870 (19)	C20—H20	0.9300
N3—H32	0.909 (19)	C21—H21	0.9300
N4—C19	1.371 (4)	C22—H22A	0.9600
N4—C22	1.430 (5)	C22—H22B	0.9600
N4—H4A	0.87 (2)	C22—H22C	0.9600
N5—C23	1.336 (3)	C23—H23	0.9300
N5—C27	1.331 (3)	C24—C23	1.373 (4)
N6—C28	1.319 (4)	C24—H24	0.9300
N6—H61	0.904 (19)	C25—C24	1.369 (4)
N6—H62	0.878 (19)	C25—H25	0.9300
C1—C2	1.488 (4)	C26—C25	1.383 (4)
C2—C3	1.393 (4)	C26—C28	1.484 (4)
C2—C7	1.371 (4)	C27—C26	1.374 (4)
C3—C4	1.362 (4)	C27—H27	0.9300
			
O1^i^—Co1—O1	180.000 (1)	C7—C6—H6	119.9
O1—Co1—O4	92.44 (9)	C2—C7—C6	121.8 (3)
O1^i^—Co1—O4	87.56 (9)	C2—C7—H7	119.1
O1—Co1—O4^i^	87.56 (9)	C6—C7—H7	119.1
O1^i^—Co1—O4^i^	92.44 (9)	N1—C8—H8A	109.5
O1—Co1—N2	89.34 (8)	N1—C8—H8B	109.5
O1^i^—Co1—N2	90.66 (8)	N1—C8—H8C	109.5
O1—Co1—N2^i^	90.66 (8)	H8A—C8—H8B	109.5
O1^i^—Co1—N2^i^	89.34 (8)	H8A—C8—H8C	109.5
O4^i^—Co1—O4	180.00 (9)	H8B—C8—H8C	109.5
O4—Co1—N2	88.05 (10)	N2—C9—C10	122.1 (3)
O4^i^—Co1—N2	91.95 (10)	N2—C9—H9	118.9
O4—Co1—N2^i^	91.95 (10)	C10—C9—H9	118.9
O4^i^—Co1—N2^i^	88.05 (10)	C9—C10—H10	120.1
N2^i^—Co1—N2	180.0	C11—C10—C9	119.8 (3)
O5^ii^—Co2—O5	180.00 (10)	C11—C10—H10	120.1
O5—Co2—O8	90.59 (8)	C10—C11—C12	119.2 (3)
O5^ii^—Co2—O8	89.41 (8)	C10—C11—H11	120.4
O5—Co2—O8^ii^	89.41 (8)	C12—C11—H11	120.4
O5^ii^—Co2—O8^ii^	90.59 (8)	C11—C12—C13	117.2 (3)
O5—Co2—N5	89.28 (8)	C11—C12—C14	125.2 (3)
O5^ii^—Co2—N5	90.72 (8)	C13—C12—C14	117.6 (2)
O5—Co2—N5^ii^	90.72 (8)	N2—C13—C12	124.2 (3)
O5^ii^—Co2—N5^ii^	89.28 (8)	N2—C13—H13	117.9
O8^ii^—Co2—O8	180.00 (12)	C12—C13—H13	117.9
O8—Co2—N5	92.47 (8)	O3—C14—N3	121.5 (3)
O8^ii^—Co2—N5	87.53 (8)	O3—C14—C12	120.8 (3)
O8—Co2—N5^ii^	87.53 (8)	N3—C14—C12	117.7 (3)
O8^ii^—Co2—N5^ii^	92.47 (8)	O5—C15—C16	117.4 (3)
N5—Co2—N5^ii^	180.0	O6—C15—O5	123.2 (3)
C1—O1—Co1	128.64 (18)	O6—C15—C16	119.4 (3)
Co1—O4—H41	134 (2)	C17—C16—C15	121.3 (3)
Co1—O4—H42	111 (3)	C21—C16—C17	117.2 (3)
H42—O4—H41	106 (3)	C21—C16—C15	121.5 (3)
C15—O5—Co2	128.33 (19)	C16—C17—H17	119.4
Co2—O8—H81	125 (2)	C18—C17—C16	121.1 (3)
Co2—O8—H82	103 (2)	C18—C17—H17	119.4
H81—O8—H82	106 (2)	C17—C18—C19	121.5 (3)
C5—N1—C8	124.7 (3)	C17—C18—H18	119.2
C5—N1—H1	111 (3)	C19—C18—H18	119.2
C8—N1—H1	124 (3)	N4—C19—C20	121.9 (3)
C9—N2—Co1	123.3 (2)	N4—C19—C18	120.3 (3)
C13—N2—Co1	119.12 (19)	C20—C19—C18	117.7 (3)
C13—N2—C9	117.5 (2)	C19—C20—H20	119.9
C14—N3—H31	118 (2)	C21—C20—C19	120.2 (3)
C14—N3—H32	124 (2)	C21—C20—H20	119.9
H31—N3—H32	118 (3)	C16—C21—H21	118.9
C19—N4—C22	124.4 (3)	C20—C21—C16	122.2 (3)
C19—N4—H4A	116 (3)	C20—C21—H21	118.9
C22—N4—H4A	119 (3)	N4—C22—H22A	109.5
C23—N5—Co2	124.23 (19)	N4—C22—H22B	109.5
C27—N5—Co2	118.79 (18)	N4—C22—H22C	109.5
C27—N5—C23	116.9 (2)	H22A—C22—H22B	109.5
C28—N6—H61	124 (2)	H22A—C22—H22C	109.5
C28—N6—H62	116 (2)	H22B—C22—H22C	109.5
H62—N6—H61	120 (3)	N5—C23—C24	122.7 (3)
O1—C1—C2	116.6 (3)	N5—C23—H23	118.7
O2—C1—O1	124.1 (3)	C24—C23—H23	118.7
O2—C1—C2	119.3 (3)	C23—C24—H24	120.2
C3—C2—C1	121.4 (3)	C25—C24—C23	119.6 (3)
C7—C2—C1	121.1 (3)	C25—C24—H24	120.2
C7—C2—C3	117.5 (3)	C24—C25—C26	118.8 (3)
C2—C3—H3	119.3	C24—C25—H25	120.6
C4—C3—C2	121.3 (3)	C26—C25—H25	120.6
C4—C3—H3	119.3	C25—C26—C28	124.6 (3)
C3—C4—C5	121.2 (3)	C27—C26—C25	117.7 (3)
C3—C4—H4	119.4	C27—C26—C28	117.8 (3)
C5—C4—H4	119.4	N5—C27—C26	124.4 (3)
N1—C5—C4	120.0 (3)	N5—C27—H27	117.8
N1—C5—C6	122.1 (4)	C26—C27—H27	117.8
C4—C5—C6	117.9 (3)	O7—C28—N6	122.2 (3)
C5—C6—H6	119.9	O7—C28—C26	119.5 (3)
C7—C6—C5	120.2 (3)	N6—C28—C26	118.3 (3)
			
O4^i^—Co1—O1—C1	36.6 (2)	C1—C2—C3—C4	177.7 (3)
O4—Co1—O1—C1	−143.4 (2)	C7—C2—C3—C4	−1.1 (4)
N2^i^—Co1—O1—C1	124.6 (2)	C1—C2—C7—C6	−177.9 (3)
N2—Co1—O1—C1	−55.4 (2)	C3—C2—C7—C6	1.0 (5)
O8^ii^—Co2—O5—C15	32.2 (2)	C2—C3—C4—C5	1.1 (5)
O8—Co2—O5—C15	−147.8 (2)	C3—C4—C5—N1	178.4 (3)
N5—Co2—O5—C15	119.8 (2)	C3—C4—C5—C6	−0.8 (5)
N5^ii^—Co2—O5—C15	−60.2 (2)	N1—C5—C6—C7	−178.6 (4)
O1^i^—Co1—N2—C13	138.0 (2)	C4—C5—C6—C7	0.6 (5)
O1—Co1—N2—C13	−42.0 (2)	C5—C6—C7—C2	−0.7 (5)
O4^i^—Co1—N2—C13	−129.5 (2)	C11—C10—C9—N2	−0.4 (5)
O4—Co1—N2—C13	50.5 (2)	C12—C11—C10—C9	−0.4 (5)
O1^i^—Co1—N2—C9	−37.7 (2)	C13—C12—C11—C10	0.4 (4)
O1—Co1—N2—C9	142.3 (2)	C14—C12—C11—C10	−179.5 (3)
O4^i^—Co1—N2—C9	54.8 (2)	C11—C12—C14—O3	170.4 (3)
O4—Co1—N2—C9	−125.2 (2)	C11—C12—C14—N3	−9.6 (4)
O5^ii^—Co2—N5—C27	33.7 (2)	C13—C12—C14—O3	−9.5 (4)
O5—Co2—N5—C27	−146.3 (2)	C13—C12—C14—N3	170.5 (3)
O8^ii^—Co2—N5—C27	−56.8 (2)	N2—C13—C12—C11	0.4 (4)
O8—Co2—N5—C27	123.2 (2)	N2—C13—C12—C14	−179.7 (3)
O5^ii^—Co2—N5—C23	−149.3 (2)	O5—C15—C16—C17	−177.9 (2)
O5—Co2—N5—C23	30.7 (2)	O5—C15—C16—C21	2.4 (4)
O8^ii^—Co2—N5—C23	120.2 (2)	O6—C15—C16—C17	2.7 (4)
O8—Co2—N5—C23	−59.8 (2)	O6—C15—C16—C21	−177.0 (3)
Co1—O1—C1—O2	−18.0 (4)	C15—C16—C17—C18	179.6 (3)
Co1—O1—C1—C2	162.07 (17)	C21—C16—C17—C18	−0.7 (4)
Co2—O5—C15—O6	−12.4 (4)	C15—C16—C21—C20	−179.6 (3)
Co2—O5—C15—C16	168.20 (17)	C17—C16—C21—C20	0.7 (5)
C8—N1—C5—C4	−179.7 (4)	C16—C17—C18—C19	0.5 (5)
C8—N1—C5—C6	−0.5 (7)	C17—C18—C19—N4	179.3 (3)
Co1—N2—C9—C10	176.9 (2)	C17—C18—C19—C20	−0.1 (5)
C13—N2—C9—C10	1.1 (4)	N4—C19—C20—C21	−179.3 (3)
Co1—N2—C13—C12	−177.1 (2)	C18—C19—C20—C21	0.1 (5)
C9—N2—C13—C12	−1.1 (4)	C19—C20—C21—C16	−0.4 (5)
C22—N4—C19—C18	−178.1 (3)	C25—C24—C23—N5	−1.1 (5)
C22—N4—C19—C20	1.3 (6)	C26—C25—C24—C23	1.4 (5)
Co2—N5—C23—C24	−177.3 (2)	C27—C26—C25—C24	−0.5 (5)
C27—N5—C23—C24	−0.2 (4)	C28—C26—C25—C24	−179.0 (3)
Co2—N5—C27—C26	178.4 (2)	C25—C26—C28—O7	−174.4 (4)
C23—N5—C27—C26	1.2 (4)	C25—C26—C28—N6	7.8 (5)
O1—C1—C2—C3	178.0 (2)	C27—C26—C28—O7	7.1 (5)
O1—C1—C2—C7	−3.1 (4)	C27—C26—C28—N6	−170.7 (3)
O2—C1—C2—C3	−1.9 (4)	N5—C27—C26—C25	−0.9 (5)
O2—C1—C2—C7	176.9 (3)	N5—C27—C26—C28	177.7 (3)

Symmetry codes: (i) −*x*+1, −*y*+1, −*z*+1; (ii) −*x*+2, −*y*+2, −*z*.

### Hydrogen-bond geometry (Å, °)

**Table:**

*D*—H···*A*	*D*—H	H···*A*	*D*···*A*	*D*—H···*A*
O4—H41···O7^iii^	0.88 (2)	1.75 (2)	2.618 (4)	173 (4)
O4—H42···O2^i^	0.89 (4)	1.86 (4)	2.644 (3)	146 (4)
O8—H81···O3^iv^	0.91 (2)	1.84 (2)	2.742 (3)	173 (3)
O8—H82···O6^ii^	0.94 (3)	1.76 (3)	2.648 (3)	156 (3)
N3—H32···O2^v^	0.91 (3)	2.04 (3)	2.939 (4)	168 (3)
N4—H4A···O4^v^	0.88 (4)	2.53 (4)	3.288 (4)	145 (3)
N6—H61···O6^vi^	0.90 (3)	2.08 (3)	2.973 (4)	169 (3)
N6—H62···O3^iii^	0.88 (3)	2.36 (3)	3.124 (4)	146 (3)

Symmetry codes: (iii) −*x*+1, −*y*+1, −*z*; (i) −*x*+1, −*y*+1, −*z*+1; (iv) *x*, *y*+1, *z*; (ii) −*x*+2, −*y*+2, −*z*; (v) −*x*+2, −*y*+1, −*z*+1; (vi) *x*−1, *y*, *z*.
